# What's your poison? Impact of individual repair capacity on the outcomes of genotoxic therapies in cancer. Part II – information content and validity of biomarkers for individual repair capacity in the assessment of outcomes of anticancer therapy

**DOI:** 10.1080/13102818.2014.902532

**Published:** 2014-05-06

**Authors:** Rumena Petkova, Pavlina Chelenkova, Elena Georgieva, Stoian Chakarov

**Affiliations:** ^a^ Scientific Technological Service (STS), Sofia, Bulgaria; ^b^ Sofia University “St. Kliment Ohridski”, Faculty of Biology, Sofia, Bulgaria

**Keywords:** DNA repair, individual repair capacity, risk, biomarkers, toxicity

## Abstract

The individual variance in the efficiency of repair of damage induced by genotoxic therapies may be an important factor in the assessment of eligibility for different anticancer treatments, the outcomes of various treatments and the therapy-associated complications, including acute and delayed toxicity and acquired drug resistance. The second part of this paper analyses the currently available information about the possibilities of using experimentally obtained knowledge about individual repair capacity for the purposes of personalised medicine and healthcare.

## Abbreviations

*CCNH*: cyclin H
*GGR*: global genome repair
*HER2*: human epidermal growth factor receptor 2
*HNPCC*: hereditary non-polyposis colorectal carcinoma
*MSH*: MutS homologue
*MLH*: MutL homologue
*NER*: nucleotide excision repair
*NHEJ*: non-homologous end joining
*TP53*: tumour protein 53 (p53)

## Individual repair capacity and eligibility for different anticancer therapies

*Remedium ante venenum non valet.*

*(Never take the antidote before the poison)*

Latin proverb

As most of the currently used anticancer therapies work by induction of DNA damage, individual capacity for DNA repair of DNA may be an important factor in the assessment of eligibility for genotoxic therapies. At present, only the most common types of tumours and the most commonly used basic therapeutic regimens have been studied with regards to eligibility for certain types of therapy, but the field is currently in development.

One of the basic factors in the assessment whether the patient is eligible for treatment with certain type of anticancer therapy is the p53 status of the tumour – specifically, whether the tumour expresses p53 or not; if it does, whether the p53 is wild-type or a cancer-specific isoform. About 50% of human tumours carry alterations in the *TP53* gene, which is usually associated with poorer prognosis for the patient (56). The *TP53* gene (or the surrounding genomic region) may be deleted or inactivated in another manner and different segments of the gene may be altered, deleted or rearranged in order to ensure cell survival even in the presence of unrepaired damage and/or genome instability. If wild-type p53 is preserved, its up-regulation may cause mass apoptosis in cancer cells, and several anticancer agents operate on this principle. For example, the histone deacetylase agent CG200745 works by stimulating the acetylation of p53 on selected lysine residues, inducing the accumulation of p53 and the subsequent transactivation of pro-apoptotic genes (37). Resveratrol, a natural antioxidant compound, also may promote apoptosis in cancer cells by activation of the p53-dependent pathway (22, 48). Of course, therapies based on p53-activation will only work if the tumour cells are capable of expression of wild-type p53. Patients with tumours that have lost the p53 expression or express a cancer-specific isoform would not be eligible for p53-based treatments, as no significant beneficial effects could be expected. The p53 status is crucially important in assessment of eligibility for different treatments in chronic lymphocytic leukemia (CLL). Five to ten per cent of the patients with CLL have a deletion of the 17p genomic region, including the *TP53* locus. The patients without deletions of 17p are eligible for genotoxic treatments (alkylating agents, e.g. cyclophosphamide, and DNA synthesis inhibitors, e.g. fludarabine), which may produce long-lasting remissions. Patients with 17p deletions, however, may benefit more from treatments other than therapies based induction of the p53-dependent pathways, such as antibodies (alemtuzumab), immunomodulators (lenalidomide), CDK inhibitors (flavopiridol) and steroids (63). Eligibility for treatment with antagonists of the MDM2 ubiquitin ligase (e.g. nutlin) may also be dependent on the expression status of wild-type p53 in tumours (54). Nutlin is not a genotoxic agent, but it works by promotion of p53 accumulation and activation of the p53-associated pathways (55).

Determination of levels of *ERCC1* expression may assist in the identification of patients eligible for therapy with platinum derivatives (13), as patients with low or indetectable levels of ERCC1 protein in tumors exhibit better responses to platinum-based chemotherapy. The C8092A polymorphism in the 3’-untranslated region of the *ERCC1* gene is associated with lower levels of ERCC1 mRNA and protein, and is likely to be a factor in eligibility for treatment with genotoxic agents. In a large study from 2008, among 25 DNA polymorphisms in genes coding for proteins of DNA repair, maintenance of genome integrity, and progression through cell cycle, several were associated with predictably poorer response in patients with advanced lung cancer treated with platinum derivatives: rs1800975 polymorphism in the 5′-untranslated region of the *XPA* gene; *XPC*ins83; *XPD* Lys751Gln; *CCNH* (cyclin H) Val270Ala (rs2266690); *RAD23B* Ala249Val (rs1805329); and *ERCC1* C8092A polymorphisms (57).

## Individual repair capacity and survival in patients with cancer

Polymorphisms in genes coding for products functioning in maintenance of genome integrity, DNA repair and/or induction of apoptosis may significantly affect response to treatment and patient survival in cancer. Better response to treatment does not always translates directly to longer patient survival, as factors other than progression of the cancer (e.g. toxic effects from the therapy) may shorten the survival. Generally, lower-than-normal capacity for DNA repair in patients with cancer is believed to be associated with better response to genotoxic treatments. It results in increased levels of therapy-induced damage in tumour cells, causing them to slow down or stop the progression in the cell cycle or reroute to apoptosis, while cells with near-normal repair capacity would repair the damage quickly, then continue proliferating. The association, however, is not that straightforward, and the field is currently in development. At present, best studied with regard to response to genotoxic therapies and patient survival are polymorphisms in the *TP53*, *XPA, XPC*, *XPD, XPG* and the *ERCC1* genes.

As it was already mentioned, *TP53* status (presence/absence of wild-type *TP53* gene copies) may be a significant factor of survival in patients with chronic lymphocytic leukemia. In 20–30% of all patients CLL may present as indolent disease, with a prolonged clinical course (up to 10–20 years) and requiring specific treatment only in the late stages or not at all. In patients with 17p deletions, however, the clinical course may be typical of an aggressive tumour, resistant to chemotherapy. Deletion of the *TP53* locus is associated with more aggressive course and, respectively, with shorter survival, in multiple myeloma (15).

The role of the common Pro72Arg polymorphism in the *TP53* gene as a survival-modifying factor in various cancers has been extensively studied, but the results so far have been, at best, contradictory. As the 72Arg allele of the *TP53* gene is associated with increased propensity to apoptosis, it could be expected that the carriers of Arg alleles (specifically, the Arg/Arg homozygotes) would be at selective advantage in cancer, as the cancer cells with Arg/Arg genotype would presumably be routed more easily to apoptosis. Indeed, patients with the Pro/Pro homozygous genotype and hereditary predisposition to nonpolyposis colon cancer exhibited earlier age of onset and a generally poorer prognosis than the carriers of Arg alleles, specifically the Arg/Arg homozygotes (28). Similar results were obtained for prostate cancer, namely, the progression from benign adenoma to carcinoma was more commonly seen in carriers of the TP53 Pro/Pro genotype (3). The Arg variant of the Pro72Arg polymorphism of *TP53* has recently been found to be associated with longer survival after conventional chemotherapy in patients with sarcoma that have retained the wildtype *TP53* gene (38). It had been demonstrated, however, that the carriership of 72Arg alleles is not an advantage in all types of tumours, as Arg/Arg carriers made the majority of patients with cervical cancer (62). Inactivation of wildtype p53 is a crucial component of the cancerous transformation of HPV-infected epithelial cells (19). The 72Arg allele of *TP53* turned out to be a preferred target for conversion into cancer-specific *TP53* variants in HPV-induced head and neck cancers, while the Pro allele was selectively inactivated or deleted (35). The same phenomenon was observed in tumours of non-viral origin, e.g. non-small-cell lung cancer (36). Retention of the Arg allele in tumour tissue may be associated with shorter survival in heterozygous Pro/Arg patients with breast cancer (6).

Carriership of the duplication variant in intron 3 of the *TP53* gene may be associated with poorer prognosis in patients with non-small-cell lung cancer (5).

Homozygous carriership of the *XPA* gene variant rs1800975 in patients with advanced lung cancer treated with platinum-based regimens is associated with shorter survival (57). Similar association has been recently demonstrated in patients with squamous carcinoma of the esophagus (59).

Genotypes containing at least one deletion allele by the intron 9 polymorphism in the *XPC* gene may be associated with poorer prognosis in patients with advanced lung cancer treated with platinum derivatives (57). The survival was longer for patients with one deletion allele compared to double del/del homozygotes. Homozygocity by the *XPC* Lys939Gln polymorphism was recently associated with shorter survival in esophageal squamous cell carcinoma (59).

Analysis of the available data indicates that the Lys751Gln polymorphism in the *XPD* gene is generally reported as not associated with significant differences in the response to chemotherapy with platinum derivatives, or patient survival (for review see [41]), albeit earlier studies indicated for shorter survival in carriers of the 751Gln allele (9). Carriership of the XPD 312Asn allele was found in the same study to be associated with poorer response to platinum-based regimens than the Asp312 variant. Carriership of the 312Asn allele in the *XPD* gene and the 399Gln allele in the *XRCC1* may be associated with shorter survival in patients with non-small-cell lung cancer treated with platinum agents (20).

A composite *XPD* genotype made of Asn at codon 312 and Gln at codon 751 of the *XPD* gene may be associated with poorer response to chemotherapy and decreased overall survival in advanced non-small-cell lung cancer (7).

Sometimes, polymorphism in one of the genes coding for products acting in DNA repair may affect the status of another repair gene. For example, carriership of the variant alleles of the *XPD* Asp312Asn and the XRCC1 Arg399Gln polymorphisms was found to be associated with higher rate of occurrence of *TP53* mutations in non-small-cell lung cancer (18, 21). This effect, however, seemed to be significant only in ever-smokers, as in patients that had never smoked the impact of the Asp312Asn polymorphism was negligibly low (17). Association between carriership of polymorphic variants of genes of DNA repair and the risk for occurrence of *TP53* gene mutations may also be observed in breast cancer, where the XPC 939 Gln/Gln, XRCC1 399 Gln/Gln and XPC 499 Ala/Ala homozygous genotypes were associated with increased risk for mutations in *TP53* (49).

Heterozygocity by the *ERCC1* T19007C polymorphism may be associated with better response (in terms of 5-year survival) in patients treated with combined radio- and chemotherapy for squamous carcinoma of the oesophagus, compared with either of the homozygous genotypes (34).

The His1104Asp variant of the *ERCC5* gene was found to be associated with poorer survival in both non-small-cell and small cell lung cancer (33). The same polymorphism was associated with better response to thalidomide therapy in refractory multiple myeloma, but this did translate in longer overall survival (12). The His46His synonymous substitution in *ERCC5* gene was reported to be associated with better clinical response to therapy with platinum derivatives in patients with non-small-cell cancer (52).

Absence or drastic reduction of ERCC1 expression (at mRNA as well as protein level) was repeatedly reported to be associated with better response to chemotherapy with platinum derivatives and increased overall survival (4, 39). Some natural compounds with alleged anticancer properties, such as emodin and curcumin, have been found to enhance the anticancer properties of cisplatin via down-regulation of the expression of ERCC1 (27, 30). The A allele of the *ERCC1* C8092A polymorphism was found to be associated with shortened survival in patients with non-small-cell lung cancer and advanced colorectal cancer, respectively, treated with platinum-based chemotherapy (40, 65). Patients on cisplatin-based chemotherapy for nasopharyngeal carcinoma with A-allele carrying genotypes for the C8092A polymorphism were shown to be at risk for faster cancer progression (11). There are, however, reports demonstrating exactly the opposite relationship between carriership of the different allelic forms of the C8092A polymorphism and patient survival. For example, the A allele of the very same C8092A polymorphism was reported by some authors to actually increase the survival rates in patients with non-small-cell lung cancer treated with platinum agents (25).

Another single-nucleotide polymorphism in the 3’- UTR of the *ERCC1* gene (Lys259Thr, rs735482) was found to be associated with longer overall survival and better response to therapy in patients with refractory multiple myeloma treated with thalidomide (12).

The C allele of the synonymous (Asn-Asn) C-to-T substitution at codon 118 of the *ERCC1* gene, also associated with differential mRNA levels, conferred longer survival in patients with advanced colorectal carcinoma treated with platinum derivatives (40). In a study from 2004, the survival of patients with non-small-cell lung cancer on combination chemotherapy with cisplatin carrying the C allele was found to be significantly longer than the T allele carriers, and specifically the T/T homozygotes (46). Interestingly, a study in patients with non-small-cell lung cancer on a docetaxel/cisplatin regimen showed the opposite association, that is, longer survival of the patients with T/T genotypes by the *ERCC1* Asn118Asn polymorphism compared to patients with C/T and C/C genotypes (24). The T allele of codon 118 ERCC1 polymorphism was found to be associated with longer progression-free survival in pancreatic cancer as well (26).

The *XPD* Lys751Gln, *XPC* Ala499Val and *XPC* Lys939Gln polymorphisms were associated with higher risk for relapse and shorter survival in patients with acute myeloid leukemia that had been placed by pre-treatment cytogenetics into the ‘intermediate’ risk group, where risk of relapse was difficult to evaluate by other methods (50).

Carriership of variant allele of the *XRCC1* Arg194Trp polymorphism may indicate better response to treatment with platinum derivatives in patients with advanced non-small-cell lung cancer (52).

## Individual repair capacity and development of drug resistance in patients with cancer

High levels of mRNA and protein of the factors of NER ERCC1 and XPD were reported to be associated with resistance to cisplatin therapy in some tumours (e.g. non-small-cell lung cancer) (29, 44). The over-expression of one of these two proteins is often associated with increased expression of the other, thus accelerating the rate of repair of iatrogenic damage in tumour cells. Carriership of the variant alleles of some of the polymorphisms of genes coding for proteins of DNA repair that are associated with decreased mRNA and protein levels – namely, *ERCC1* (C8092A)*, XRCC1* (Arg399Gln) and *XPD* (Lys751Gln), may confer lower risk for development of resistance to platinum-based therapy in some tumours (non-small-cell lung cancer, gastric carcinoma) (25, 42). The effect may be dose-dependent in carriers of one or two copies of the allele (53).

Deletion of both somatic *TP53* copies in multiple myeloma is a predictor for resistance to genotoxic therapy with 6-mercaptopurine (pyrimidine analogue) or melphalan (16).

The genome of a cancer cell is changeable, and some traits may be lost, while others may be newly acquired. Resistance to an anticancer drug usually develops in the course of treatment with the drug. So far there have been several experimental proofs that some cancer cells may restore the activity of previously inactive or weakly active repair proteins by mutagenesis, resulting in development of resistance to a drug to which the tumour was initially sensitive. Experiments with mouse models of breast cancer carrying frameshift mutations in the *BRCA1* gene show that some tumours that initially were sensitive to anticancer therapy (specifically, cisplatin) may be capable of restoring the functionality of *BRCA1* (8). This is achieved by error-prone copying during replication, introducing new mutations in the gene – usually, small deletions and insertions, resulting in restoring the reading frame and producing almost full-length BRCA1 protein, which ultimately produces resistance to cisplatin (8, 45). A similar reversion of mutated inactive *BRCA2* allele to a functional allele, conferring resistance to a genotoxic drug to which the tumour was initially sensitive, was described to have occurred *in vivo*, in a tumour from a patient with Fanconi anemia complementation group D1 and acute myelogenous leukemia (23).

## Individual repair capacity and the risk of toxicity of anticancer therapies

Heterozygous carriership of mutations associated with the phenotype of ataxia-telangiectasia or ‘neutral’ polymorphisms in the *ATM* gene may be associated with high toxicity in patients treated with ionising radiation (2). Three polymorphisms in the *ATM* gene – 126713G-to-A, 111G-to-A (rs189037) and G5557A, were found to be associated with increased risk for severe radiation pneumonitis in patients with lung cancer treated with radiotherapy (58, 64). Increased risk for acute skin and haematological toxicity after genotoxic therapies may be dependent on carriership of the Lys939Gln polymorphism in the *XPC* gene; Lys751Gln and Asp312Asn polymorphisms in the *XPD* gene; Arg194Trp, Arg280His and Arg399Gln polymorphisms in the *XRCC1* gene; and the Asp148Glu polymorphism in the gene coding for the apurinic/apyrimidinic endonuclease APE1, an enzyme functioning in base excision repair and mismatch repair (10, 47, 57). Carriership of the Arg399Gln polymorphism in the *XRCC1* gene and the Thr241Met polymorphism in the *XRCC3* gene may predispose to radiation-induced subcutaneous fibrosis and telangiectasias after radiotherapy (1).

Studies of the effects of multiple (more than 4–5 per study) polymorphisms on the risk of an associated disease or condition, response to therapy and/or associated adverse effects are still rare in the specialized literature. The results from one extensive study for association of carriership of DNA polymorphisms and therapy-associated toxicity in patients with esophageal cancer treated with ionising radiation were published in 2011. Significant association with severe (grade 3-4) toxicity (myelosuppression/dysphagia) was identified for 12 out of 21 polymorphisms in genes coding for products functioning in the maintenance of genome integrity and/or DNA repair and the control of the progression in the cell cycle (60). The 12 polymorphisms may be grouped by function as follows: mismatch repair: *MSH6* Gly39Glu; nucleotide excision repair: the rs1800975 polymorphism in the 5′-untranslated region of the *XPA* gene; *XPC* Lys939Gln; *XPD* Lys751Gly; and the more rare synonymous substitution *XPD* Asp711Asp; *ERCC1* C8092A; specifically for transcription-coupled repair: *ERCC6* (*CSB*) Met1097Val; base excision repair: *XRCC1* Arg399Gln; control of the progression in the cell cycle: *CCNH* Val270Ala; repair of double-strand breaks: *XRCC2* 5′-untranslated region polymorphism rs6464268 (homologous recombination) and the synonymous Asp568Asp polymorphism in the gene *LIG4* (non-homologous end joining); damage-related signaling: *BRCA1* Pro871Leu (rs799719). The highest odds ratio (>8) was identified for the 5’-UTR rs6464268 in the *XRCC2* gene. This was not unexpected, as double-strand breaks are among the major types of damage caused by ionising radiation. As XRCC2 protein functions in repair of double-strand breaks by homologous recombination, it could be expected that even subtle deficiencies in this repair pathway would have significant impact on repair of therapy-inflicted damage (31, 32). For the polymorphisms *XPD* Asp312Asn, *RAD23B* Ala249Val, *APE1* Asp148Glu (rs1130409) and *PARP1* Val762Ala (rs1136410), the association with risk of radiation-induced toxicity was estimated to be weak.

The synonymous polymorphisms Asp568Asp in the *LIG4* gene and Asp711Asp in the *XPD* gene, the 5′-untranslated region polymorphism in *XRCC3* and the Val219Ile polymorphism in the coding sequence of the *MLH1* gene were found to be associated with late rectal or bladder toxicity in patients treated with radiotherapy for prostate cancer (14).

It would be logical to assume that polymorphic gene variants associated with superior survival after genotoxic treatments may also be factors in the constitution of the risk for treatment-associated toxicity. Tumour cells with lower capacity for DNA repair rapidly accumulate unrepaired DNA damage, resulting in cell cycle arrest and/or apoptosis. The healthy cells in the patient, however, carry the same genotype, and have the same lower-than-normal capacity for DNA repair, therefore, they would also suffer more damage from the genotoxic treatments. The relationship is more complex, however, and alleles that confer lower repair capacity are not always associated with severe toxic effects. For example, among patients with non-small-cell lung cancer treated with platinum derivatives, increased risk for high-grade gastrointestinal toxicity was not seen in carriers of the C allele of the *ERCC1* gene (associated with lower *ERCC1* transcript stability and lower protein levels), but in carriers of the A allele (51). Recently, it was reported that the A allele in the C8092A polymorphism was related to higher levels of unrepaired DNA adducts in human lymphocytes (61). Apparently, the effects of a DNA polymorphism or haplotype on the toxicity profile of genotoxic therapies are dependent on additional factors, including phenotypic factors. Obesity for example, is the single factor that is associated with increased risk for acute toxicity in all types of anticancer treatments (43). This is most likely related to the fact that most treatments are administered as a dose per kg body weight or square meter of body surface, therefore, in large patients, a large dose may be needed, which is potentially associated with severe toxic effects.

## Conclusions

Modern biomedical science develops rapidly, and more and newer therapies are being developed every day, providing if not a cure, then at least a considerable improvement in patient survival and quality of life. This is especially valid in cancer, a very common disease that was not that long ago considered incurable and uniformly fatal. Cancer is currently believed to result from accumulation of unrepaired damage in DNA, and treatment of cancer is very often based on inflicting DNA damage in order to reduce the proliferative potential of tumour cells. Natural variance in the individual capacity for repair of damage in DNA may play significant role in the risk for development of cancer. When cancer has already developed, individual repair capacity may modulate various aspects of its course, such as whether the patient would benefit from this or that therapy; the uneventful and overall survival of the patient; whether there are modifiable environmental factors that may increase the chances for better therapeutic response and/or lower treatment-related toxicity; the risk for development of resistance to antitumour drugs in the course of treatment; and the possible adverse effects of genotoxic therapies. Knowledge about the individual specificities of capacity for repair of DNA damage provides a base for making informed decisions about potential changes in lifestyle, selection of treatments best suited to the particular patient, prognostication about survival and anticipation and management of potential complications.
